# A Systematic Review on Lean Applications’ in Emergency Departments

**DOI:** 10.3390/healthcare9060763

**Published:** 2021-06-19

**Authors:** Davenilcio Luiz Souza, André Luis Korzenowski, Michelle McGaha Alvarado, João Henrique Sperafico, Andres Eberhard Friedl Ackermann, Taciana Mareth, Annibal José Scavarda

**Affiliations:** 1 Industrial & Systems Engineering Department, Polytechnic School, University of Vale do Rio dos Sinos, São Leopoldo 93022-750, RS, Brazil; davenilciol@edu.unisinos.br (D.L.S.); joao.sperafico@vs.unimed.com.br (J.H.S.); andresackemann@edu.unisinos.br (A.E.F.A.); 2 Accounting Department, School of Management and Business, University of Vale do Rio dos Sinos, Porto Alegre 91330-002, RS, Brazil; tmareth@unisinos.br; 3 Industrial & Systems Engineering Department, Herbert Wertheim College of Engineering, University of Florida, Gainesville, FL 32611, USA; alvarado.m@ufl.edu; 4 Department of Production Engineering, Center for Exact Sciences and Technology, Federal University of the State of Rio de Janeiro, Rio de Janeiro 22290-255, RJ, Brazil; annibal.scavarda@unirio.br

**Keywords:** Lean, healthcare, emergency departments, systematic literature review, PRISMA

## Abstract

This article presents the state of the art of Lean principles applied in Emergency Departments through a systematic literature review. Our article extends previous work found in the literature to respond to the following questions: (i) What research problems in emergency departments can Lean principles help overcome? (ii) What Lean approaches and tools are used most often in this environment? (iii) What are the results and benefits obtained by these practices? and (iv) What research opportunities appear as gaps in the current state of the art on the subject? A six-step systematic review was performed following the guidance of the PRISMA method. The review analysis identified six main research problems where Lean was applied in Emergency Departments: (i) High Waiting Time and High Length of Hospital Stay; (ii) Health Safety; (iii) Process redesign; (iv) Management and Lessons Learned; (v) High Patient Flow; (vi) Cost Analysis. The six research problems’ main approaches identified were Lean Thinking, Multidisciplinary, Statistics, and Six Sigma. The leading Lean tools and methodologies were VSM, Teamwork, DMAIC, and Kaizen. The main benefits of applying Lean Principles were (a) reductions in waiting time, costs, length of hospital stay, patient flow, and procedure times; and (b) improvements in patient satisfaction, efficiency, productivity, standardization, relationships, safety, quality, and cost savings. Multidisciplinary integration of managers and work teams often yields good results. Finally, this study identifies knowledge gaps and new opportunities to study Lean best practices in healthcare organizations.

## 1. Introduction

Emergency Departments (ED) are places that offer accident and emergency care services at different severity levels. Because these departments are complex systems, there is no default template to improve performance [1]. It is common to see overcrowding and workloads that exceed the availability of resources [2], which results in high waiting times, delays in acute treatments, and increased lengths of hospital stays [3]. These issues impact the quality of care and clinical outcomes, increasing the rate of adverse events and hospital mortality [4]. The challenge for healthcare companies is to find appropriate management solutions that improve efficiency, productivity, and quality of performance [3]. In this context, hospital costs and patient and worker satisfaction are affected [5,6].

Factors causing an imbalance of care in ED are usually related to inadequate services. For example, patients in non-urgent situations can seek treatment from outpatient clinics [4]. The ED is an embedded environment with interdependent upstream relationships where downstream processes impact patient flow across the entire hospital system [7].

Health systems are composed of a network of service providers, interconnected between the public and private environments. This system presents several challenges regarding public policies, financing constraints, infrastructure, and human resources [8]. Thus, management and decision-making precision are essential, so it does not impact the quality of care [1]. In this way, it is necessary to find viable solutions to improve resource efficiency in EDs through layout redesign, resource capacity, or revised process flow. The detection of bottlenecks in the emergency processes is one indicator of the need for improvement in emergency health services [9].

Enterprises of health services may improve the quality and capacity by applying Lean principles [3,10,11]. Lean has two pillars: autonomy and Just in Time. Lean operates on the principle of generating value for the customer, society, and economy, in addition to reducing waste [12,13]. The first reports of Lean application for improving patient flow came in 2002 [14]. Lean principles provide EDs with an excellent approach to continuous process improvement. Several of the studies presented aim to reduce waste and improve emergency care processes [15].

Implementation of Lean principles in healthcare can improve care capacity by as much as 30% and reduce patient waiting time by 50% [16]. However, the interaction between working groups with Lean is variable [17], which indicates that the successful implementation of Lean depends on the leaders’ commitment. Other elements for the success of Lean methodology in an ED is the ability to change, developing a clear mapping of processes and the introduction of small improvements that are sustainable in the long term [3]. In complex environments such as health, it is vital to combine Lean’s continuous improvement with innovative practices to strengthen the implementation of improvement projects [15]. More rigorous studies are needed to determine Lean benefits in healthcare [18,19], including those focused on the implementation process, which can serve as a roadmap for new applications. The management tools applied to healthcare processes are difficult to implement due to absence of standardization and repeatable indicators to measure the quality of care [20]. Otherwise, Ref. [21] points out that a limited number of studies address shortcomings in the ED, but a growing trend utilizes hybrid methods that involve simulation, Lean techniques, and quality improvement. The use of an integrated methodology is desirable because Lean methodology is very suitable for increasing the effectiveness of the process, while Six Sigma tools increase the efficiency of the process [11]. There are studies that apply Six Sigma DMAIC methodology [11,22] and Lean Six Sigma management tools [20], which indicate improvement of healthcare performance after implementation. The integrated use of both methodologies for the analysis of processes allows the improvement of the overall performance. Similar contributions are expected to expand the current body of research for the ED and increase utilization of these hybrid approaches.

This article seeks to present the current state of the art through a systematic review of the literature on applying Lean principles in EDs. We have identified 56 articles published between 2013 and 2020. Previous systematic review articles suggest new approaches involving Lean principles in EDs [18,19,21]. The main contribution of this article lies in the extent of previous review articles identified in literature by answering the following questions: (i) What research problems in emergency departments can Lean principles help overcome? (ii) What Lean approaches and tools are used most often in this environment? (iii) What are the results and benefits obtained by these practices? and (iv) What research opportunities appear as gaps in the current state of the art on the subject? In this systematic review, articles are classified by research problem into six main groups. Additionally, nine usual approaches and eleven main tools and methods are noted among the included studies. The Lean principles used in emergency departments are identified, grouped by similarity, and organized. Finally, the main improvements and benefits of applying these tools are also listed for each study. The remainder of this article appears as follows: In Section 2, the systematic review methodology is presented. Section 3 reveals the study environment characterization. Section 4 features the research problems, approaches, and tools associated with Lean. In Section 5, we discuss the results and benefits. Finally, in Section 6, conclusions and research opportunities are presented.

## 2. Materials and Methods

The methodology adopted is a systematic literature review. This methodology groups previous work on a specific topic, promoting the identification, evaluation, and study interpretation in a given area by analyzing concepts and practices. Next, we summarize the subjects’ state of the art and address research gaps [23,24]. We conduct the research based on the a six-step systematic literature review (SLR) approach adopted by [25]: (i). planning and formulation of the problem, (ii). search in the literature, (iii). collection and evaluation of data, (iv). analysis and synthesis of data, (v). interpretation, and (vi). results presentation. The working method for the search in the literature step follows the guidance of the PRISMA Group [26].

**Figure 1:**
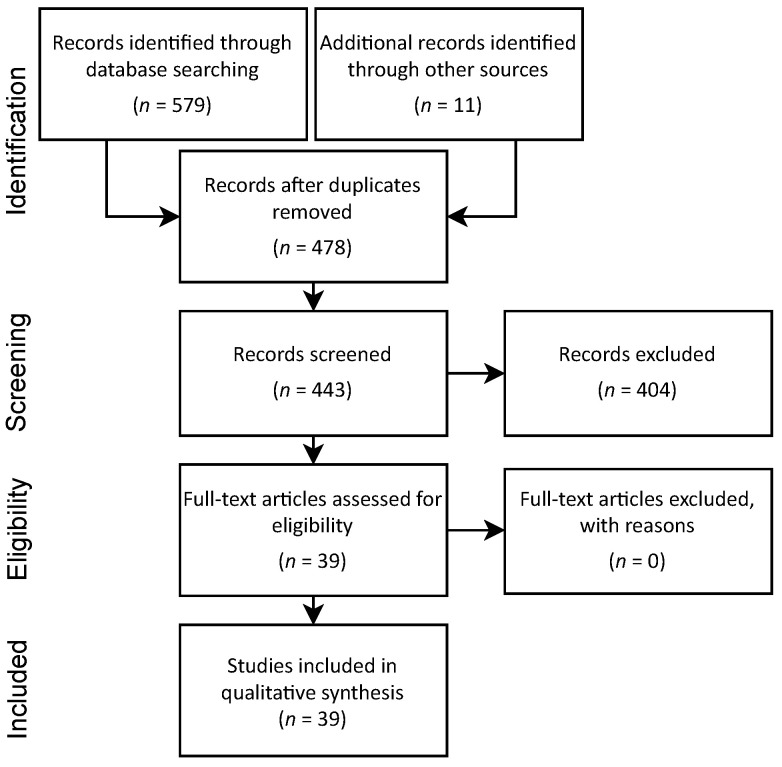
Flow diagram of systematic literature review.

First, we selected databases containing scientific journals related to the research theme: Emerald, Elsevier, Web of Science, Science Direct, Scopus, Sage, Wiley, and EBSCO. In the second step, the terms “Lean” and “Lean Health Care” and “Emergency Department” were inserted into search engines of each source for titles, abstracts, and keywords. Figure 1 summarizes the articles and the systematic review steps.

From 2013 to 2020, the search found 579 articles plus 11 articles from other references resulting in 590 peer-reviewed articles written in English. In the third step, we applied the exclusion criteria. There were 112 duplicates removed, resulting in 478 articles screened by abstract. Then, 392 papers were removed after additional methodological content analysis (superficial content and gray literature), leaving 86 papers remaining for eligibility. Gray literature is “scientific newsletters, reports, working papers, theses, government documents, newsletters, brochures, congress proceedings, and other publications distributed free of charge, available by subscription” [27].

Three authors conducted a full-text review in the fourth step for the 86 eligible manuscripts. Additional exclusion resulted in 30 articles removed because they did not mention the Emergency Department, leaving 56 documents in the final selection for synthesis in step six. The authors’ interactive review process was provided significant agreement for the content analysis. In addition to the general characteristics of the articles identified and the number of articles per year, the articles were classified according to the following criteria: research problems, methodological approaches, tools and methods, and benefits in terms of reductions and improvements. Analysis of criteria aimed to point out the associations between these and the research problems identified in the review. The organization of language into categories increased in importance given the large volume of information available [28]. In this context, this article adopts the following definitions for the elements addressed in its criteria. a. Methodological approach: the concept attributed to the research methods adopted in the researched articles; b. Lean approaches: the concepts attributed to the means that supported the application of the tools and methods; and c. Tools and methods: these were treated together to facilitate the grouping of resources for implementing the listed improvements.

Table 1 summarizes the characteristics of the 56 studies analyzed in this systematic review based on whether the approach was qualitative or quantitative, whether the study was theoretical or empirical, and the research method type (case study, modeling, etc.).

Most of the studies used a qualitative approach (n=33/56) or theoretical–empirical type (n=32/56). As for the research method, the majority were characterized as case studies (n=37/56). It is noteworthy that practices such as simulation and experimentation were rarely used. This systematic review focused on studies applying Lean techniques, which usually describe empirical results without further quantitative treatment.

## 3. Emergency Department Patient Flow

Worldwide, the health system is composed of a network of public and private service providers. Whether public or private, emergency services consist of 24-h, 7-day-a-week care. Generally, the patient flow goes through the following steps: registration, screening, first medical care, exams, medication administration and/or observation room, hospitalization and/or medical discharge [1].

Emergency Departments pertain to a wide range of medical circumstances. Consequently, there is a considerable variation in the number and type of processes and resources required in each patient’s care flow. However, as cited by [16], there are five common steps according to the literature, as follows:

- Screening: performed by a nursing professional, brief assessment of patient clinical conditions regarding severity to define care priority;
- Medical care: physical examination and interview to understand the main complaint;
- Initial diagnosis: actions based on the primary complaint, oral medication or other modality, and auxiliary services (as needed);
- Intermediate diagnosis: additional service result evaluation and/or medication administration;
- Final diagnosis: hospitalization or medical discharge.

Due to physicians and nursing teams’ complexity, patients’ service limitations include lack of beds, inflexible paper-based systems, precautionary isolation, delays in cleaning, excessive bed dependence for patients under observation or hospitalization, and unsatisfactory diagnoses or discharge instructions [29].

## 4. Research Problems, Approaches, and Applied Tools

We found seven articles on average per year in the search period, focusing on emergency departments Lean implementation. We can highlight the years 2014, 2015, and 2016 with the highest publications of 10, 9, and 9 articles respectively, but there was a downward trend in the subsequent four years, with six publications on average per year.

The primary research problems addressed in the articles were Management and Lessons Learned and Process Redesign, which were prevalent in fifteen papers each. High Waiting Time and High Length of Hospital Stay were the research problems in thirteen articles, followed by High Patient Flow in nine articles, Health Safety in three articles, and Cost analysis in one article.

Next, the research team performed a content analysis on selected articles to describe the research problems, the approaches, and applied tools on Lean implementation. Table 2 summarizes findings of content analysis. All of the research problems are described in detail later in this section.

The Lean Thinking approach was used in all 56 articles. Multidisciplinary, which refers to various functions such as clinicians, cardiologists, nurses, anesthesiologists, nursing assistants, pharmacists, and administrative staff, working together evaluating, identifying improvement actions, and developing diagnostic analysis, was the second leading approach observed in 28 articles. Statistical analysis and Six Sigma support Lean applications in 20 and 17 papers, respectively.

VSM and Teamwork were the most commonly used tools, appearing 29 times each, indicating that these two tools have been most viable for Lean principles implementation in Emergency Departments. Data analysis occurred 15 times, Kaizen events 13 times, Standardization and DMAIC 12 times each, followed by Visual Management (VM) 11 times, PDCA 6 times, and finally 5S, Spagguetty, and PDSA 5 times each. Note that the frequencies represent the number of times Lean tools and methods appear in the 56 articles.

In the following subsections, the research problems are further described, along with the approaches and tools and methods used to solve and achieve the desired results for each type of research problem. The discussion emphasizes the Multidisciplinary approaches in line with Lean Thinking principles, Six Sigma, and Statistics, among others, to reach the best results.

### 4.1. Management and Lessons Learned

Lean Thinking is evolving in health research with gains in quality, service efficiency, productivity, satisfaction, and safety for patients and healthcare professionals. Magalhães et al. [30] emphasized looking for the best scientific knowledge in health, and stated that teamwork development promotes process standardization, reduces costs, and decreases length of hospital stay. Workers were empowered to propose and implement ideas and solve problems. The primary technical, administrative, and clinical sectors were involved in routine standardization, focusing on emergency care, achieving 47%, 38%, and 16% of improvements, respectively. On average, 72% of enhancements related to perceived problems.

The Kaizen model has a high degree of problem identification and solution propositions, but a low degree in the implementations [31]. Crema realized that in a study of 22 clinical risk management project results, around 68% involved resource optimization and improvement of activities to increase efficiency and productivity, including cost and time reduction in routine execution [32].

Practitioners accepted Lean principles when they had more involvement in using the Lean Thinking and Six Sigma approach than what was reported in the literature. According to [33], the reasons were the combination of national policy, local clinical environment, and professional-design emergency medicine.

Regarding the Lean Thinking and Statistics approach, two authors conducted systematic literature review studies. Lean Thinking highlighted implementation in 27 studies. The results showed waiting time reduction in an Emergency Department (*p*-value < 0.05), improvement in material inventory management in the operating room, and reduction in 30-day mortality rates [34]. Moraros et al. [18] did not find consistency in Lean implementations in the health area from an international study. Despite this, positive results relating to improved processes were recognized.

Two studies applied the Lean Thinking and Multidisciplinary approach. Rotteau et al. [35] highlighted the key factors in implementing Lean: (i) organizational readiness; (ii) managers visibly prioritizing and supporting initiative and engagement; (iii) a clear understanding of tasks, results, and the environment; (iv) multi-media communication, held continuously to engage and inform teams; and (v) a sustainable plan that specifies those responsible for the processes and necessary changes [35]. Adopted as a philosophy and work model by health leaders, Lean is presented by [36] on six bases: (i) continuous improvement attitude; (ii) value creation; (iii) purpose unity; (iv) respect for front-line workers; (v) visual tracking; and (vi) flexibility. Electronic controls were implemented with improved attendance, waiting time improvement by 28%, hospitalization costs reduction by 25%, and significant work environment improvements [36].

The Lean Thinking, Multidisciplinary, and Six Sigma approach highlights a multidisciplinary group activity invested in the success of a new four-day protocol, called Rapid Improvement Event (RIE), in the Emergency Department, dealing with alcohol dependence at a hospital health safety network. Sankoff et al. [37] affirm that Lean is a valuable principle for addressing complex and systemic processes. Average hospitalization time decreased from 3.6 days to 2.6 days, and savings improved by 2 million dollars per year.

The Lean Thinking, Multidisciplinary, Six Sigma, and Statistics approach have highlighted Lean Six Sigma as a model management improvement. As a main result, Dyas et al. [38] highlight a dedicated Scanner installation that reduced the average processing time in the emergency room from 113 to 58 min.

According to [17], approaches combining Lean Thinking, Multidisciplinary, and Statistics presented significant results in wards (*p* = 0.11) but were not significant in the Emergency Department (*p* = 0.02). Different levels of commitment among health teams showed success and failure in implementing Lean in each location.

In an approach to Lean Thinking, Multidisciplinary, and specifically Business Process Management (BPM) [40], highlight the teamwork integration, focusing on high-level projects in processes standardization. To avoiding patient falls, patients’ locomotion improved Emergency Department quality indicators. There were reductions in costs, inpatient time, and infection time alert and gains in efficiency, satisfaction, professional’s safety, and patient safety.

The socio-technical field influenced [39] to utilize Lean Thinking and the Psychosocial Approach via active staff participation in Ward I, resulting in a stable and well-organized Lean implementation. Nursing II obtained partial results, but results deteriorated in the Emergency Department. A regression analysis indicated that the results were significant in the Emergency Department (*p* = 0.02) and in Ward II (*p* = 0.04), and not significant in Ward I (*p* = 0.11). The unsatisfactory results in Ward II and the Emergency Department were due to the lack of employee need perception while implementing Lean principles, which was influenced by declining interest among senior management. The psychosocial work climate refers to a positive commitment of all staff and is negative when neglected, mainly due to its leadership. In the same vein, Alnajem et al. [41] highlight the influence of senior management on encouraging positive relations between health professionals, patients, and suppliers. Multisdisciplinary reinforces improvement in Teamwork and standardization of activities.

Finally, the Lean Thinking and Business Planning Improvement (BPI) approaches emphasize Lean and agile actions categories derived from the hospital’s literature. Olsson and Aronsson [42] found that most agile efforts are reactive, indicating a lack of measure prevention. The Leagile approach is three combinations derived from the hospital’s literature (customer focus, small advances, and advances validation).

### 4.2. Process Redesign

Based on the Lean Thinking approach and the DEMATEL decision-making method in a public hospital Emergency Department, work teams applied Value Stream Mapping and visual management to identify relevant factors and reduce patient waiting time and length of hospital stay. The data analysis improved efficiency in service due to the equipment availability. These results influenced the patients’ perception of value in care [43].

Supported by Lean Thinking and Six Sigma, the workplace redesign reduced variability, caused changes in culture, team integration, and gains in patient self-esteem [44]. Leaders influence the work team on their walks through the workplace. Training activities and events to identify improvements such as Kaizen encourage other spontaneous activities by service personnel. The practice of 5S and visual management facilitates the activities’ organization and procedures standardization in the Emergency Department [45].

The Lean Thinking and Statistics approaches were implemented in the Emergency Department’s linear layout using Value Stream Mapping (VSM) and simulation. The application reduced the average patients’ waiting time by 50% with service level gain and personnel nurses relocation by 30% [16]. In a district emergency department, a work team conducted a pre–post-intervention study. During the intervention phase, patients were screened using the single-layer system. The intervention reduced the overall hospital stay from 106 to 85 min. The subgroup analysis showed a reduction in the length of hospital stay for non-critical cases from 72 to 45 min [46].

Four authors referenced Lean Thinking and Multidisciplinary. A framework created the evaluation methodology to quantify health institutions’ readiness and guide practitioners in the Lean project’s implementation [51]. Health managers look for ways to improve organizational performance in continuous improvement activities. Lean Healthcare is continuously challenged and used successfully to investigate, design, and strengthen process systems to deliver health services [47]. A multidisciplinary team of management and frontline staff defined values-based outcome measures, mapped the current processes, and redesigned them to achieve the ideal. The Lean methods foster interdisciplinary teams and problem-solving across departments. There is one approach in Veterans Affairs Emergency Department that can be used to address systemic factors and contributors to reduce crowding and improve care for Veterans [50,52].

There is evidence that Lean design application brings substantial improvements in people skills, working in the laboratory, and the reliability needed to determine the most appropriate changes. Improved flow of samples, technicians, supplies, and patient information reduced 187 km and eight days of unnecessary walking per year [48]. Lean is a practical methodology for improving processes in emergency departments. As a result, nine projects were implemented in hospitals with success, improvement in communication, geographic assignment by specialty, and visual management [49].

A large hospital implemented the process redesign project by Lean Thinking, Multidisciplinary, and Six Sigma approach. A study with non-probabilistic samples by interviews was conducted to improve Emergency Department efficiency. There was a low agreement among the participants regarding the quality of care and the project’s success. The project did not eliminate existing divisions between doctors, managers, and physicians. Tension was perceived by the team, hindering patient care agility. A coordinated effort is needed in each hospital to improve patients’ access to Emergency Care [53].

To identify the main problems of the Emergency Departments, Ref. [21] carried out a literature review where Lean and Six Sigma principles, Statistics, and Multidisciplinary were the means for identifying the five main problems: excessive length of hospital stay, long waiting times, excessive patient flow, overcrowding, and leaving without being seen. The most used tools were: Value Stream Mapping, the PDCA cycle, and data analysis. The survey revealed the long waiting time by the limited capacity of beds, staff unavailability, and the inadequate layout in the Emergency Department. These factors permeate patients’ lack of understanding of the Emergency Department’s purpose. To promote solutions to these problems, Lean principles integrated with the Voice of the Customer and the Voice of the Process perspectives were used [54].

Based on a Lean Thinking, Multidisciplinary, and Resilience Engineering approach, an empirical study was carried out on the Emergency Department flow in an intensive care unit. The study gave rise to a framework with eight project propositions for implementation in this unit. Both the framework and its proposals can contribute to the design of socio-technical systems that are both safe and efficient [55].

### 4.3. High Waiting Time and High Length of Hospital Stay

Recall that high waiting time and high length of hospital stay was the primary research problem in nine studies. In one of them, Kane et al. implemented Lean Thinking to support a specific local system of a healthcare unit, leading to the creation of the Stanford Operating System (SOS) [56]. Using Kaizen rapid improvements, the study achieved significant gains such as patient satisfaction, reduction in waiting time, and standardized activities [56]. With Lean Thinking, it was also possible to obtain reductions in the average time of care from emergency to hospitalization and patient discharge. In the lead time of patient flow, there was a significant reduction in costs [57].

One article reorganized patients’ flow based on Lean principles to improve capacity indicators and patient flow performance in the Emergency Department without adding costs. The outcomes included reducing waiting time for procedures by 40% and a gain of 40% capacity within one hour of discharge [10,62].

Combining the Lean Thinking and Six Sigma approach as a quantitative analysis resource, it was possible to identify gains in efficiency and quality of blood tests. One study achieved a mean reduction of 30% in the time of hemogram analysis, a 50% reduction in the number of vials used for testing, a 50% decrease in unusual or extra specimens, and a 90% reduction of samples from the Department of Emergency without requests for analysis.

Furthermore, accuracy in results improved due to error elimination caused by laboratory test repetition [60]. Relating Lean Thinking to Statistics can also reduce waiting times, as demonstrated by a quantitative analysis on administrative data that achieved a 50% reduction in the Emergency Department waiting time [61].

Lean in Healthcare is inspired by Lean Thinking to identify and minimize waste in healthcare institutions. According to the case study in an Emergency Department in the Silesia Province, the most common adverse event is the excessive waiting time for medical service. Value Stream Mapping (VSM) was successfully applied to reduce waiting time in all stages of medical care to implement process standardization by identifying and then eliminating waste [58].

A multidisciplinary team applied Lean principles to reduce length of hospital stay. The development of standard work management was fundamental in maintaining results in the Emergency Department. Service units have developed day-to-day management (e.g., Visual management) to reinforce, evaluate, and refine standardized work [59].

Design Event Simulation and Lean Thinking were applied to increase the number of treated patients and reduce the length of hospital stay without compromising the quality of services and patient safety. The institution organized work teams to share responsibilities at each Emergency Department patient level. In this way, the study proposed a successful project to reduce patients’ length of hospital stay in a Canadian Emergency Department. Design of Experiments (DOE) was carried out to determine optimal resource number at each shift throughout day and week. The specialists and the modeling team analyzed the simulated model. They agreed that it was as close to the actual patient flow as possible, and then experiments were performed to reduce patients’ waiting time and standardize it [63].

In the Canadian rural context, SurgeCon is a simulation platform created in conjunction with Lean principles to improve the average waiting time of the Emergency Department in community hospitals. A multidisciplinary team performed by physicians and nurses obtained significant results in developing and implementing improvements in the Emergency Department’s inefficiencies. The intervention targeted the organization of the Emergency Department: workflow, action-based management, and a patient-centered environment. A decision-making process, improving communication between the team and managers, set expectations for everyone in the Emergency Department by assigning roles [67].

A Lean Thinking, Six Sigma, and Statistics approach was implemented to reduce the length of hospital stay by 30% over three months. Additionally, the number of patients without treatment decreased from 6.5% to 0.3%, and patient satisfaction improved from 24% to 89.9%. These results put the Emergency Department at the top level (1%) of hospitals nationwide [64].

Two studies used the Lean Thinking, Multidisciplinary, and Statistics approach. Uspal et al. [65] found a significant reduction in the mean length of hospital stay of pediatric patients with psychiatric complaints and the best perception of Emergency Department care staff. Finally, Ref. [66] applied these approaches in the largest and most influential oncology unit in Spain, which led to the first steps implementing a telephone remote sorting and scheduling system to reduce patients’ displacement with limited mobility.

### 4.4. High Patient Flow

The Lean Thinking approach is connected by [68,69]. Combining Lean Thinking approach and Health Information System (HIS) as a tool, Ker et al. [68] suggest that Kaizen events are excellent for minimizing chaos and disorder in the outpatient surgery unit and reducing time and costs related to the Emergency Department’s patient flow. Shakoor et al. [69] used teamwork and Value Stream Mapping (VSM) in the trauma and rapid response environment in the Emergency Department to analyze overcrowding data and redistribute the patient arrival flow. Teamwork applied Takt Time to standardize room occupancy predictions.

According to a Lean Thinking and Multidisciplinary [19] report, a human-centered approach, top management support, standardization, teamwork allocation, and local context analysis via Value Stream Mapping are crucial for success.

Combining Lean Thinking, Multidisciplinary, and Six Sigma approaches, the academic area of healthcare highlights the need to redesign the sequential flow of attendance by integration of specialties. Rapid Process Optimization (RPO) provided time gains in several departments’ activity, improved patient flow and service performance, and reduced costs [70]. Lean principles can reduce turnaround times for Emergency Departments and contribute to better regulation compliance, improved quality of care to the patient, and promote team involvement in process awareness to diffuse Lean culture [71].

WIth Lean Thinking, Multidisciplinary, Six Sigma, and a Statistics approach, [73] identified a top-down implementation based on the Axiomatic Design of Health Care evidence. Value Stream Mapping (VSM) grouped patients with identical characteristics into families to obtain a homogeneous value flows through holistic hospital patient flow optimization and system complexity reduction.

Lean Thinking and Statistics approaches demonstrate improvements in Emergency Department flow after multimodal intervention on overcrowding and patient flow in a Dutch trauma center. Hours of crowding decreased significantly, as did the patients’ length of hospital stay. This finding led to the organization of a 5-day Lean project within the radiology department, focusing on improved turnaround time for radiography [72].

A Lean Thinking and Six Sigma approach at Saudi health centers focused on “patient in first” to develop performance improvement. The unit was named the Performance Improvement Unit (PIU) to improve the patients’ flow by redesigning the care process. The implementation results were promising but eventually stalled due to lack of support and low team engagement (staff, hospital unit managers, and the Ministry of Health) [74].

Finally, Theory of Constraints (TOC) five steps and Value Stream Mapping (VSM) are associated with the Lean Thinking approach as a tool. According to [75], these resources identify and eliminate placements that do not add value. During each stage of the patient’s journey, the mean times were compared between two groups, which were divided according to a four-hour time frame and disproportionate delays. Significance test calculations indicated that a 75% reduction in time patients were delayed due to bottlenecks [75].

### 4.5. Health Safety

Three papers used combinations of Lean Thinking, Multidisciplinary, and Six Sigma to address health safety. The studies improved compliance with legal requirements, team training in problem-solving skills, standardization of care, and collaborative team actions. The multidisciplinary team approach found that the project’s real value was to understand that achieving health safety is a journey of never-ending improvements. It is not achieved by merely getting the title of Black or Green Belt [76]. Furthermore, positive impacts were perceived by the university hospital staff and by the community on organizational culture, open communication, team-valued self-esteem, and patient safety [77]. Lean and Six Sigma projects sped up the patients’ diagnosis with chest pain in an adult emergency environment. The objectives included multidisciplinary teams to optimize flow and reduce waste between patients’ arrival and electrocardiogram execution. The door to electrocardiogram protocol implementation generated significant patient safety improvement [78].

### 4.6. Cost Analysis

The cost analysis research utilizes Lean Thinking, Six Sigma, and Statistics approaches to develop a cost-benefit analysis. The low-complexity model created by the simplified activity-based costing approach provides a useful tool for selecting, prioritizing, and validating process improvement projects in Emergency Departments and other areas involving different diagnoses. One study used these tools and models to identify cost inefficiencies and evaluate the costs of underlying process activities [79].

## 5. Benefits of Lean Application In Emergency Departments

The articles’ content analysis highlights the primary outcomes and benefits of Lean practices implemented in Emergency Departments. Table 3 presents the main results and gains found in each identified research problem, grouped by loss reduction items and performance improvement items.

**Figure 2:**
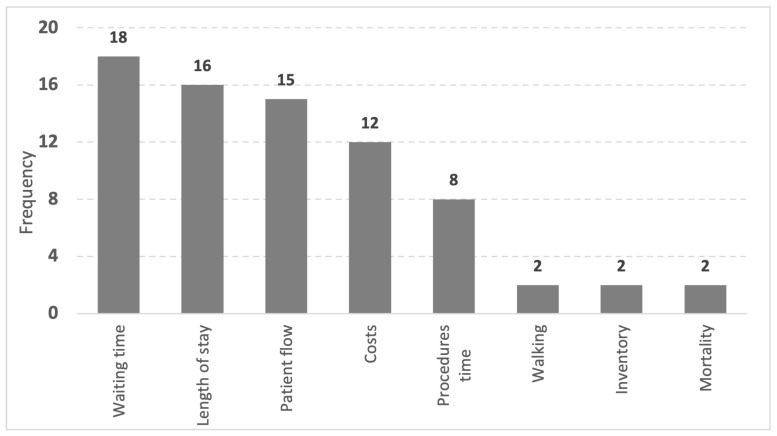
Main reductions identified in literature after Lean implementation in ED.

Figure 2 summarizes the frequency of reductions obtained, with waiting time being the most frequent reduction in nine articles. Waiting time reduction is the most significant benefit for Emergency Departments. Important waiting times reduction were from 78 to 38 min (>50%) by [16] and 28% by [36]. Waiting time reduction positively affects patient satisfaction and improves related problems such as overcrowding, lack of beds, and mortality rate. VSM is the appropriate and easy-to-use tool to identify excess patient waiting time.

Reduction in costs and length of hospital stay is also meaningful. Cost reduction (e.g., 25%) is by loss elimination, activities, and services that do not add value to stakeholders [36]. Length of hospital stay reduction (e.g., 3.6 days to 2.6 days) increases resource availability such as physical space, beds, and time for professionals to provide patient care [37].

The patient flow improvement provides comfort and satisfaction and prevents unnecessary fatigue in sensitive patients. Another benefit is the reduction in procedure times (e.g., 40%), which can reduce patient discomfort and improve service performance (e.g., 40%) capacity within one hour of discharge [10,62].

The reduction in professional movement avoids the feeling of fatigue in non-valued-added walks to care, in particular, the 187 km reduction in walking, equivalent to 8 days of lost activities per year [48].

Inventory medicine reduction brings tangible results, reducing costs, enabling the allocated resources to maintain the necessary medicine stock in a balanced way. It also brings intangible benefits in controls such as identifying and locating stock quantities, avoiding shortages, administration errors, and loss of expired medication [34].

**Figure 3:**
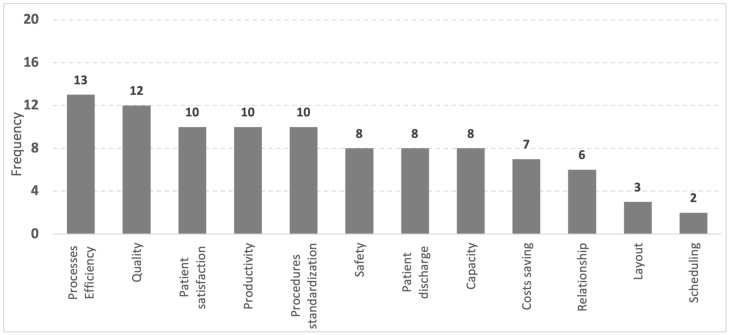
Main improvements identified in the literature after Lean implementation in ED.

The mortality reduction in 30 and 3 days is significant because it emphasizes preserving patient’s lives due to the implementation of preventive actions [18]. The Lean adoption improvements result in concrete benefits, providing quantitative and qualitative results. The level of success depends on the organization’s objectives and teamwork. Figure 3 summarizes the number of and the main improvements identified.

Patient satisfaction, ranging from 24% to 89.9% [56] and the 40% improvements in 1-hour patient discharge [57], process efficiency increasing from 54.8% to 88.5% [16], productivity improvement of 68% [30], and reduction of the average scanning time from 113 to 58 min [38] by adopting a specific scanner are examples of improvement s that bring significant benefits. Patient satisfaction rises and perceived quality increases through agile and safer processes.

Under better resource management, efficiency and productivity gains can improve waiting time and length of hospital stay. Procedure standardization, another improvement reported in studies, reduces variability, avoids errors, and gives stability to processes, making them safer, more efficient, and more productive.

Improving relationships at work, despite being intangible, is one of the most prominently successful outcomes of Lean implementation. This success depends on people’s engagement level with processes and their integration with other stakeholders such as managers, physicians, nursing teams, pharmacy, and support staff.

High levels of employee engagement (e.g., 72%) show significant results in outpatient department performance, quality, and health professionals satisfaction, which consequently increases patient satisfaction [31]. There are psychosocial environmental impacts on Lean successful implementation [39]. Lean and TOC integration with other approaches in project implementation and process improvement brings knowledge to the health organizations’ intellectual capital [75]. Patients’ and healthcare workers’ safety improvements in Emergency Departments are also featured. In some studies, successful cost-saving approaches removed unnecessary expenditure of USD 2 million per year [37] and produced useful model cost loss analysis [79]. Patients discharge has also been improved by as much as 40% [62]. Remote scheduling avoids aggravating oncology patients’ clinical condition creating reductions in displacement and waiting time [65,66]. Implementing a new layout is another benefit that positively impacts the organization’s activities and patient flow [16].

## 6. Conclusions and Research Opportunities

Studies show that a successful and sustainable deployment involves developing the culture of continuous improvement to establish a suitable and perennial environment for Lean application [10,62]. Multidisciplinary team members need to be trained on Lean concepts and tools and be encouraged to propose and implement ideas for problem-solving [31]. Implementation of Lean approaches in pilot environments before full-scale or generalized implementation is essential to avoid team frustration in unsuccessful projects [61]. Continuous and efficient communication also helps in spreading this culture [35].

The most present research problems in this environment are waiting time, length of hospital stay, patient safety, process redesign, patient flow, resource management, lessons learned, and cost analysis. Usually, Lean is presented together with another approach, highlighting the complexity of developing workable solutions with a single procedure. Besides Lean Thinking, the Multidisciplinary approach appears as one of the most relevant techniques for projects in complex environments like Emergency Departments.

From the articles’ review, it was possible to observe the challenges in Emergency Departments such as waiting time, patient flow, bed unavailability, difficulty in screening, and length of hospital stay. Lean overcame these challenges. However, in the article from [74], solutions to improve patient flow management were disrupted by the lack of managers’ engagement and teams in project implementation. In the article from [53], a weak relationship between Managers and Physicians about work divisions also reduced the performance speed of care.

This study provided an overview of Lean application in Emergency Departments based on a systematic review. It was possible to identify a rise in Lean in health verified by gains of quality, service efficiency, productivity, satisfaction, and patients’ and professionals’ safety and health. According to Lean’s goal of adding value to the client, society, and economy, we show several studies reducing waste in health services. Lean and complementary approaches such as Six Sigma can increase capacity and reduce waiting time, length of hospital stay, and service costs. We should also consider the interaction between working groups, which it is imperative for Lean implementation. The leaders’ commitment to teamwork progress is necessary, and the continuous improvement culture is an essential element.

Given the research objectives and delimitation, we suggest future work to develop frameworks using Multidisciplinary approaches with Lean and other methods in an integrated way. Similar research in distinct departments may complement the health literature review. It is also important to highlight that in 2020, we were affected by the coronavirus disease pandemic (COVID-19). Some studies on the impact of the Pandemic in emergency departments can be highlighted [80,81,82,83]. Although these studies focus on the main trans-disciplinary aspects of team preparation, based on the specifics of each emergency center, the discussion goes beyond any type of benefit that the emergency departments may have enjoyed from the previous implementation of Lean principles. The verification through an efficiency study of the existence of differences between the emergency departments of hospitals that implemented and did not implement tools and Lean methods in their operation emerges as a current and relevant research opportunity. Note that the screening process needs a detailed analysis of the value flow and can use tools such as VSM, for example, to improve their results. Finally, value attributes to patients and physicians also promote Lean tools in health.

## Figures and Tables

**Table 1:** Studies characteristics.

Characteristics	Classification	Frequency
Methodological Approaches	Qualitative	33
	Qualitative/Quantitative	19
	Quantitative	4
Type of Study	Theoretical-Empirical	32
	Empirical	20
	Theoretical	4
Research Method	Case Study	37
	Literature Review	11
	Survey	5
	Modeling	3

**Table 2:** Summary of research problems, approaches, and tools and methods by authors.

Research Problem	Authors	Approach	Tools and Methods
	[30,31,32]	LT, HLM	Process Control; Kaizen; PDSA; CRM
	[33]	LT, Six Sigma	DMAIC
	[18,34]	LT, Statistic	Data Analysis; Standardization
	[35,36]	LT, Md	Teamwork; Standardization; VSM; PDSA; VM
Management and Lessons Learned	[37]	LT, Md, Six Sigma	Kaizen; DMAIC; A3; PDCA; Teamwork
	[38]	LT, Md, Six Sigma, Statistic	VSM; DMAIC; Kaizen; Control Chart; Teamwork
	[39]	LT, Statistic, Md	Teamwork; MASP; VM; 5S; Data Analysis
	[40]	LT, Md, BPM	Service; Teamwork
	[17,41]	LT, Psycho-social	Service; VSM; Teamwork; Standardization
	[42]	LT, BPI	VSM; Lean-Agile
	[43]	LT	VSM; Teamwork; Data Analysis; VM
	[44,45]	LT, Six Sigma	Kaizen; DMAIC; Teamwork; VM; 5S; Standardization
	[16,46]	LT, Statistic	VSM; WIP; Simulation; Data Analysis
Process Redesign	[47,48,49,50,51,52]	LT, Md	VSM; Kaizen; Teamwork; Spaghetti; Standardization
	[53]	LT, Md, Six Sigma	VSM; DMAIC; Teamwork;
	[21,54]	LT, Statistic, Six Sigma, BPM	VSM; PDCA; Data Analysis; PDSA; VM; Standardization
	[55]	LT, Md, RE	VSM; Kaizen; Teamwork; PDCA
	[56,57,58]	LT	VSM; 5S; Kaizen; Spaghetti; ADM; Check Sheet; Standardization
	[59]	LT, Md	Teamwork; VM; Standardization
High Waiting Time and High Length of Hospital Stay	[60]	LT, Six Sigma	VSM; DMAIC; PDCA; Kaizen; 5S; Poka Yoke; VM
	[10,61,62,63]	LT, Statistic	VSM; Kaizen; Data Analysis; Cohort; Standardization
	[64]	LT, Statistic, Six Sigma	VSM; Kaizen; DMAIC; PDCA
	[65,66,67]	LT, Statistic, Md	VSM; Control Chart; Teamwork; ADM; Data Analysis
	[68,69]	LT	Kaizen; HIS; Teamwork; VSM; Data Analysis; Standardization
	[19]	LT, Md	VSM; Teamwork; Standardization
	[70,71]	LT, Md, Six Sigma	DMAIC; RPO; 5S; PDSA; Teamwork
High Patient Flow	[72]	LT, Statistic	Teamwork; Data Analysis
	[73]	LT, Six Sigma, Md, Statistic	VSM; DMAIC; Teamwork; Data Analysis; Design Axiomatic
	[74]	LT, Six Sigma	VSM; DMAIC; VM
	[75]	LT, TOC	VSM; Steps of TOC
	[76]	LT, Md, Six Sigma	VSM, DMAIC, VM, Teamwork, PDCA
Health Safety	[77]	LT, Statistic, Md	PDSA; Teamwork; Data Analysis
	[78]	LT, Md, Statistic, Six Sigma	Data Analysis; Standardization; Teamwork
Cost Analysis	[79]	LT, Statistic, Six Sigma	VSM; DMAIC; Spaghetti; SIPOC; ABC

Approach Abbreviations: (LT) Lean Thinking; (Md) Multidisciplinary; (RE) Resilience Engineering; (HLM) Health Lean Management; (BPM) Business Process Management; (BPI) Business Process Improvement; (TOC) Theory of Constraints. Tools and Methods Abbreviations: (ADM) Active Daily Management; (A3) A3 Report; (VM) Visual Management; (5S) Seiri, Seiton, Seis¯ o, Seiketsu, and Shitsuke; (CRM) Clinical Risk Management; (HIS) Health Information System; (RPO) Rapid Process Optimization; (SIPOC) Suppliers, Inputs, Processes, Outputs, Customers; (ABC) Activity-Based Costing; (VSM) Value-stream Mapping; (DMAIC) Define, Measure, Analyze, Improve, Control; (PDCA) Plan, Do, Check, Act; (PDSA) Plan, Do, Study, Act; (MASP) Method, Analysis, Solve, Problem.

**Table 3:** Benefits of Lean applications.

Research Problem	Results/Benefits	
Management and Lessons Learned	Reduction	Waiting time, costs, Length of hospital stay [30,31,32]; Waiting time, Inventory, Mortality rates at 30 days and 3 days [18,34]; Waiting time −28%, Costs −25% [35,36]; Length of hospital stay from 3.6 days to 2.6 days [37]; Waiting time, Costs, Length of hospital stay [40]; Waiting time, Patient flow [41].
	Improvement	Productivity +68%, efficiency, Process standardization, Quality service, Satisfaction and safety to patients and professionals, Improvement suggestions given by 72% of the employees [30,31,32,33]; The Lean implementations suggest new studies [18,34]; Capacity of service +10% [35,36]; Savings of U$ 2 million per year [37]; Average processing time Scanner from 113 min to 58 min [38]; Significant results in wards I and II and not significant in ED, Differences in teams performances [39]; Productivity, Efficiency, Quality, Satisfaction and safety to patients and professionals, Standardization [40]; Psico-ssocial influence in successful Lean implementation [17]; Lean-Agile solutions [42]; Processes Efficiency, Procedures Standardization, Relationship [41].
Process Redesign	Reduction	Waiting time from 78 to 38 min [16]; Walking −187 km = 8 work days/year [47,48,49,51]; Waiting time from 34.7 to 22.1 min, Length of hospital stay from 163.2 to 146.3 min [52]; Waiting time −48%, Patient flow, Length of hospital stay from 8.7 to 6.4 h, Costs [21]; Length of hospital stay, Patient flow [46]; Costs, Length of hospital stay, Patient flow, Walking [45]; Waiting time, Length of hospital stay, Patient flow, Inventory [43]; Waiting time, Costs, Patient flow, Procedure times, Mortality [54]; Waiting time, Patient flow [50].
	Improvement	Procedures standardization [44]; New Lay-out, Efficiency from 54.8% to 88.5% [16]; Processes [47,48,49,51]; Weak relationship between Managers and Physicians about work divisions [53]; Framework, Design social-technical system, efficiency, safer place [55]; Relationship, Layout, Patient Discharge from 161.8 to 156.6 min [52]; Patient Satisfaction, Patient Discharge [21]; Processes Efficiency, Productivity [46]; Patient Satisfaction, Efficiency, Productivity, Standardization, Quality, Cost Saving, Patient Discharge, Capacity [45]; Patient Satisfaction, Efficiency, Productivity, Safety, Quality, Cost Saving [43]; Patient Satisfaction, Processes Efficiency, Productivity, Procedures Standardization, Relationship, Safety, Quality, Cost Saving, Discharge, Layout, Capacity [54]; Patient Satisfaction, Processes Efficiency, safety, Quality, Patient Discharge, Capacity from 25 to 34 patients [50].
High Waiting Time and High Length of Hospital Stay	Reduction	Waiting time, Lead time and costs [56,57]; Time exam execution −40%, Waiting time for results [60]; Length of hospital stay −30% [64]; Procedure times [65,66]; Length of hospital stay from 199.4 to 134.4 min [67]; Procedure times from 104.3 to 42.2 min [67]; Waiting time [58]; Waiting time, Costs, Length of hospital stay, Patient flow, Procedure times [63]; Length of hospital stay from 8.7 to 6.4 h = 26.4% [59].
	Improvement	Patient satisfaction [56,57]; +40% of patient discharge in 1 h [10,61,62]; Patient satisfaction from 24% to 90% [64]; Remote scheduling [65,66]; Patient satisfaction, Capacity 25.7%, Processes Efficiency [67]; Processes Efficiency, Procedures Standardization, Quality, Patient Discharge [58]; Patient Satisfaction, Efficiency, Procedures Standardization, Safety, Quality, Cost Saving, Patient Discharge, Scheduling, Layout, Capacity [63]; Procedures Standardization [59].
High Patient Flow	Reduction	Patient flow, Costs [68]; Waiting time, Patient flow Redesign [70]; Patient flow [73]; Waiting time, Patient flow [75]; Waiting time, Patient flow [71]; Length of hospital stay from 167 to 154 min, Patient flow, Procedure times [72]; Waiting time, Costs, Length of hospital stay [69]; Length of hospital stay, Cost Saving [19].
	Improvement	Performance indicators, Specialities Integration [70]; Axiomatic design according Lean Health Care [73]; Performance Improvement Unit (PIU) weak integration between teams and managers [74]; Integration of Lean and TOC principles [75]; Processes Efficiency, Patient Satisfaction, Relationship, Quality [71]; Quality [72]; Processes Efficiency, Quality, Cost Saving, Patient Discharge, Capacity [69]; Productivity, Patient Satisfaction, Capacity [19].
Health Safety	Reduction	Procedure times from 17 to 7 min, Waiting time, Costs, Patient flow, Mortality [78].
	Improvement	Procedures standardization [76]; Patient satisfaction [77]; Procedures Standardization, Safety, Quality, Cost Saving [78].
Cost Analysis	Improvement	Cost model creation make easier to identify waste in expenses [79].

## Data Availability

The data of researched articles and classification presented in this study are available on request from the corresponding author. The researched articles and books are available in the journals/editor where they were originally published.
